# Transferrin-Conjugated pH-Responsive γ-Cyclodextrin Nanoparticles for Antitumoral Topotecan Delivery

**DOI:** 10.3390/pharmaceutics12111109

**Published:** 2020-11-18

**Authors:** Seonyoung Yoon, Yoonyoung Kim, Yu Seok Youn, Kyung Taek Oh, Dongin Kim, Eun Seong Lee

**Affiliations:** 1 Department of Biotechnology, The Catholic University of Korea, 43 Jibong-ro, Bucheon-si, Gyeonggi-do 14662, Korea; y_seonyoung@naver.com (S.Y.); rladbsdud727@catholic.ac.kr (Y.K.); 2 School of Pharmacy, Sungkyunkwan University, 2066 Seobu-ro, Jangan-gu, Suwon-si, Gyeonggi-do 16419, Korea; ysyoun@skku.edu; 3 College of Pharmacy, Chung-Ang University, 221 Heukseok dong, Dongjak-gu, Seoul 06974, Korea; kyungoh@cau.ac.kr; 4 Department of Pharmaceutical Sciences, College of Pharmacy, University of Oklahoma Health Sciences Center, 1110 N Stonewall Ave, Oklahoma City, OK 73117, USA; Dongin-Kim@ouhsc.edu; 5 Department of Biomedical-Chemical Engineering, The Catholic University of Korea, 43 Jibong-ro, Bucheon-si, Gyeonggi-do 14662, Korea

**Keywords:** pH-Responsive γ-cyclodextrin, tumor-targeted drug delivery system, transferrin, topotecan

## Abstract

In this study, we developed γ-cyclodextrin-based multifunctional nanoparticles (NPs) for tumor-targeted therapy. The NPs were self-assembled using a γ-cyclodextrin (γCD) coupled with phenylacetic acid (PA), 2,3-dimethylmaleic anhydride (DMA), poly(ethylene glycol) (PEG), and transferrin (Tf), termed γCDP-(DMA/PEG-Tf) NPs. These γCDP-(DMA/PEG-Tf) NPs are effective in entrapping topotecan (TPT, as a model antitumor drug) resulting from the ionic interaction between pH-responsive DMA and TPT or the host–guest interaction between γCDP and TPT. More importantly, the γCDP-(DMA/PEG-Tf) NPs can induce ionic repulsion at an endosomal pH (~6.0) resulting from the chemical detachment of DMA from γCDP, which is followed by extensive TPT release. We demonstrated that γCDP-(DMA/PEG-Tf) NPs led to a significant increase in cellular uptake and MDA-MB-231 tumor cell death. In vivo animal studies using an MDA-MB-231 tumor xenografted mice model supported the finding that γCDP-(DMA/PEG-Tf) NPs are effective carriers of TPT to Tf receptor-positive MDA-MB-231 tumor cells, promoting drug uptake into the tumors through the Tf ligand-mediated endocytic pathway and increasing their toxicity due to DMA-mediated cytosolic TPT delivery.

## 1. Introduction

For successful chemotherapy, developing multifunctional drug delivery systems to improve the therapeutic effect while minimizing the side effects of drugs has been widely conducted worldwide [1,2,3]. In particular, recent intensive research on stimulus-responsive drug-carrying systems that induce explosive drug release by reacting sensitively to changes in pH, temperature, and localized enzyme expression in specific tissues has been widely conducted [1,3,4,5]. Interestingly, among these physical environmental factors, the pH-stimulus factor is remarkable because it is a special feature that appears inside the cellular endosome/lysosome and around solid tumors [1,2,3,4,5]. For example, the weakly acidic pH condition (~6.8) of the tumor-surrounding environment formed by the anaerobic metabolism of tumor tissues can be targeted using pH-responsive polymeric carrier systems [1,2,3,4,5]. Furthermore, if the particles absorbed by tumor cells through endocytosis have an endosomal-escaping ability, more effective cytosolic drug delivery and synergistic therapeutic effects can be expected. However, the particles without an endosomal-escaping ability cannot move to the cytoplasm, are usually exocytosized, or eventually move to the major degradative compartments (lysosomes) of cells [1,2,3,4,5,6,7]. Therefore, recent research has concentrated on exploiting stimulus-responsive multifunctional drug-carrying platforms that effectively recognize site-spatial tumor environments and exhibit excellent endosomal-escaping ability [7,8,9,10,11,12,13,14,15]. Indeed, considering the potential in vitro/in vivo antitumor efficiency of these systems, the design and development of pH-responsive endosomolytic drug-carrying particles may guarantee relatively high antitumor efficiency, even if tumor environments possess an inherently unpredictable complexity [1,2,3,4,5,16].

**Figure 1:**
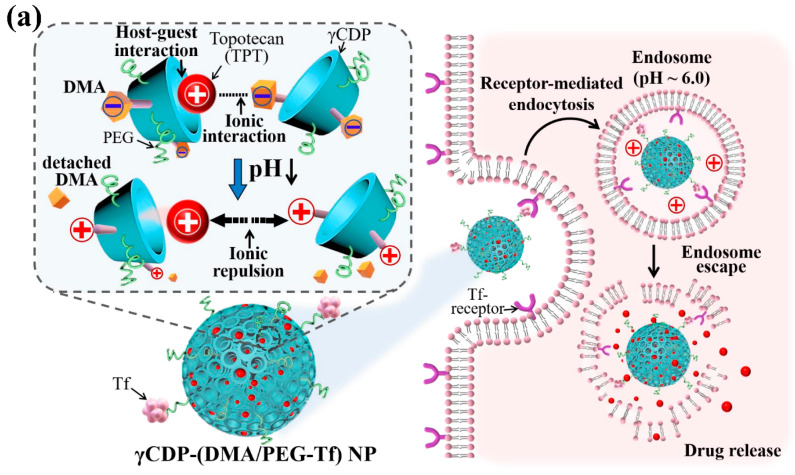

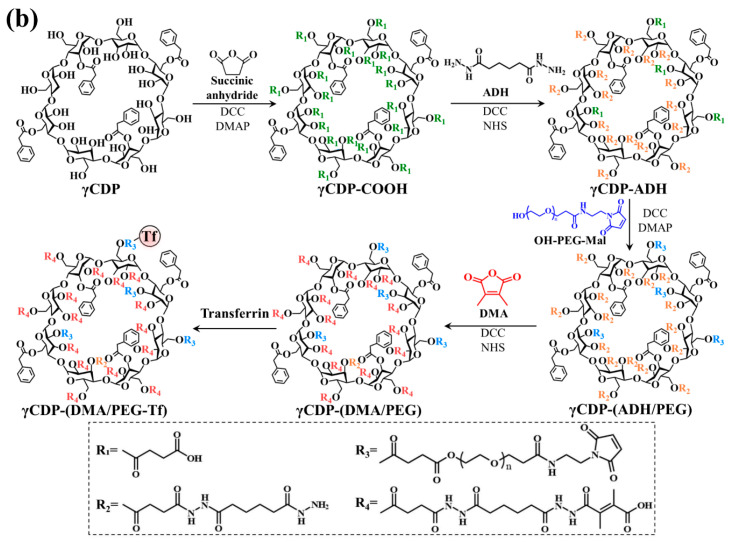
(**a**) The schematic illustration of transferrin-conjugated pH-responsive γ-cyclodextrin (γCD) nanoparticles coupled with phenylacetic acid (PA), 2,3-dimethylmaleic anhydride (DMA), poly(ethylene glycol) (PEG), and transferrin (Tf) (γCDP-(DMA/PEG-Tf) NPs). (**b**) The synthesis scheme of γCDP-(DMA/PEG-Tf).

In this study, we developed pH-responsive γ-cyclodextrin (γCD)-based nanoparticles (NPs) for delivery of topotecan (TPT, as a model antitumor drug) (Figure 1a). First, we prepared γ-cyclodextrin (γCD) conjugated with phenylacetic acid (PA, as a hydrophobic moiety) [16,17,18,19,20,21], denoted as γCDP. The resulting γCDP was subsequently coupled with 2,3-dimethylmaleic anhydride (DMA, as a pH-responsive moiety) [22,23], poly(ethylene glycol) (PEG, as a colloidal stabilizer) [6,7,24], and transferrin (Tf, as a target protein for Tf receptor-positive tumor cells) [2,6,25,26,27] (Figure 1b), denoted as γCDP-(DMA/PEG-Tf). We also fabricated γCDP-(DMA/PEG-Tf) NPs through the self-assembly of PEG and Tf on a nanoparticular shell and DMA-conjugated γCDP on a porous core (Figure 1a). It is well known that γCD with a toroid-like structure is biocompatible, biodegradable, and nontoxic, and it exhibits an attractive affinity for small molecules (e.g., TPT) [8,16,17,18]. In addition, DMA coupled with γCDP can electrostatically interact with cationic TPT molecules [15], resulting in leading high TPT encapsulation in porous channels of γCDP. However, DMA can be chemically detached from γCDP in response to a reduction in pH from 7.4 to 6.5 (i.e., endosomal pH) [22,23], resulting in encouraging ionic repulsion between γCDP and TPT and then activating TPT release to the cell cytoplasm (Figure 1a). We anticipate that this pH-responsive behavior of γCDP-(DMA/PEG-Tf) NPs will be significant as a promising antitumor platform for efficient tumor killing. In this study, we specifically investigated the pH-responsive properties, antitumor drug (TPT) release profiles, and in vitro/in vivo antitumor efficiency of γCDP-(DMA/PEG-Tf) NPs to evaluate their pharmaceutical potential.

## 2. Materials and Methods

### 2.1. Materials

γ-Cyclodextrin (γCD), succinic anhydride (SA), *N*,*N*′-dicyclohexyl carbodiimide (DCC), 4-dimethylaminopyridine (DMAP), *N*,*N*-dimethylformamide (DMF), dimethylsulfoxide (DMSO), phenylacetic acid (PA), adipic acid dihydrazide (ADH), *N*-hydroxysuccinimide (NHS), dichloromethane (DCM), 2,3-dimethylmaleic anhydride (DMA), transferrin (Tf), *N*-(2-aminoethyl maleimide) (Mal), triethylamine (TEA), pyridine, triton X-100, and formaldehyde were purchased from Sigma-Aldrich (St. Louis, MO, USA). OH–PEG–COOH (Mn = 2 kDa) was purchased from Biochempeg Scientific Inc. (Watertown, MA, USA). A micro bicinchoninic acid (BCA)^TM^ protein assay kit was purchased from Thermo Scientific Inc. (Waltham, MA, USA). Topotecan hydrochloride (TPT) was purchased from US Pharmacopeia (North bethesda, MD, USA). Roswell Park Memorial Institute (RPMI)-1640 medium, fetal bovine serum (FBS), phosphate-buffered saline (PBS), penicillin, trypsin, ethylenediaminetetraacetic acid (EDTA), and streptomycin were purchased from Welgene Inc. (Seoul, Korea). Cell Counting Kit-8 (CCK-8) was purchased from Dojindo Molecular Technologies Inc. (Rockville, MD, USA). The Annexin V–fluorescein isothiocyanate (FITC) apoptosis detection kit was purchased from BD Pharmagen™ (San Jose, CA, USA). Chlorin e6 (Ce6) was purchased from Frontier Scientific Inc. (Logan, UT, USA). γCDP (γCD conjugated with PA) and Mal-functionalized PEG were synthesized as described in detail our previous reports [16,27].

### 2.2. Preparation of γCDP-(DMA/PEG-Tf)

To synthesize the γCDP-(DMA/PEG-Tf), γCDP (1 g) was first reacted with SA (0.8 g) in DMSO (20 mL) containing pyridine (1 mL), DCC (2.3 g), DMAP (1.2 g), and TEA (1 mL) at 25 °C for 3 days, producing carboxylated γCDP (γCDP–COOH). The resulting solution was dialyzed using a membrane tube (Spectra/Por^®^ MWCO 1 kDa) against fresh DMSO for 2 days and deionized water for 2 days [28,29,30,31]. After lyophilizing, γCDP–COOH (0.5 g) was reacted with ADH (0.8 g) in DMF (50 mL) containing DCC (1.4 g), NHS (1 g), pyridine (1 mL), and TEA (1 mL) at 25 °C for 3 days. The resulting solution was purified through dialysis tubes (Spectra/Por^®^ MWCO 1 kDa) against fresh DMSO for 2 days and deionized water for 2 days, and then lyophilized. The residual carboxyl groups of the γCDP-ADH (0.2 g) reacted with the hydroxy group of OH-PEG-Mal (0.6 g) in DMSO (20 mL) containing DMAP (0.05 g), DCC (0.1 g), pyridine (1 mL), and TEA (1 mL) at 25 °C for 3 days. The resulting solution was dialyzed using preswollen membrane tubes (Spectra/Por^®^ MWCO 2 kDa) in fresh DMSO for 2 days and deionized water for 2 days and then lyophilized [24,27,28,29,30,31,32], producing γCDP-(ADH/PEG). The amine groups of γCDP-(ADH/PEG) were subsequently coupled with DMA (0.3 g) in DMF (15 mL) containing DCC (0.2 g), NHS (0.1 g), and TEA (1 mL) at 25 °C for 3 days. The resulting solution was dialyzed using preswollen dialysis membrane tubes (Spectra/Por^®^ MWCO 2 kDa) against fresh DMSO for 2 days and 5 mM sodium tetraborate solution for 2 days to remove the non-reacted chemicals, producing γCDP-(DMA/PEG) (Figure 1b). The Ce6 dye-tagging process for fluorescent in vivo studies was performed through the chemical reaction between γCDP-(DMA/PEG) (200 mg) and Ce6 dye (10 mg, preactivated using ADH (5 mg) in DMF (10 mL) containing TEA (200 μL), DCC (7 mg), and NHS (5 mg) at 25 °C for 1 day) in DMSO at 25 °C for 2 days as described in our previous reports [28,29]. Next, the Mal group in γCDP-(DMA/PEG) (or Ce6 dye-tagged γCDP-(DMA/PEG), 10 mg) was reacted with the thiol group of the Tf (1 mg) during dialysis (Spectra/Por^®^ MWCO 10 kDa) in 150 mM PBS (pH 7.4, 10 mL), producing γCDP-(DMA/PEG-Tf) NPs (or Ce6 dye-tagged γCDP-(DMA/PEG-Tf) NPs). Here, the Tf concentration in NPs was analyzed using a micro BCA^TM^ protein assay for a supernatant of the NP solution ultracentrifuged at 25,000 rpm for 20 min [25,26]. In addition, γCDP-(PEG-Tf) NPs were synthesized under the same conditions without DMA. Finally, we prepared four types of NPs: γCDP-(DMA/PEG-Tf) NPs, γCDP-(DMA/PEG) NPs (without Tf), γCDP-(PEG-Tf) NPs, and γCDP-(PEG) NPs (without Tf).

### 2.3. TPT Loading

The NPs (γCDP-(DMA/PEG-Tf) NPs, γCDP-(DMA/PEG) NPs, γCDP-(PEG-Tf) NPs, and γCDP-(PEG) NPs: 20 mg) were mixed with TPT (10 mg) in deionized water for 1 day. The resulting solution was dialyzed and purified through an ultracentrifugation process at 20,000 rpm for 30 min, and then lyophilized. The amount of TPT entrapped in NPs was calculated after measuring the TPT fluorescence intensity of the supernatant using a microplate reader (Bio-Tek, Winooski, VT, USA) at λ_excitation_ of 400 nm and λ_emission_ of 530 nm. The loading efficiency (%) of TPT in each NP sample was defined as the weight percentage of TPT in the NP sample relative to the initially feeding amount of TPT. The loading content (%) of TPT in each NP sample was calculated as the weight percentage of TPT in the NPs [25,26].

### 2.4. Characterization of NPs

The particle size and zeta potential of each NP sample (0.1 mg/mL) was measured using a Zetasizer 3000 instrument (Malvern Instruments, Malvern, UK) after being stabilized in 150 mM PBS (pH 7.4, 6.5, or 6.0) at 25 °C for 4 h. The morphology of each NP sample was confirmed using a field-emission scanning electron microscopy (FE-SEM, Hitach S-400, Fukuoka, Japan) after being stabilized in 150 mM PBS (pH 7.4, 6.5, or 6.0) at 25 °C for 4 h [32,33,34,35,36,37].

### 2.5. In Vitro TPT Release Test

Each NP sample (1 mg/mL) was added to a dialysis membrane tube (Spectra/Por MWCO 50 kDa) and immersed in 15 mL of fresh PBS (150 mM, pH 7.4, 6.5, or 6.0). The membrane tubes were incubated in a shaking water bath (100 rpm) at 37 °C for 48 h. At each time point, the external PBS solution of the dialysis tubes was replaced with a fresh PBS solution. The amount of TPT released from the NP sample was determined after measuring the fluorescence intensity of the PBS solution using a microplate reader (Bio-Tek, Winooski, VT, USA) at λ_excitation_ of 400 nm and λ_emission_ of 530 nm [15,23,24,25].

### 2.6. Cell Culture

Human breast carcinoma MDA-MB-231 cells and Chinese hamster ovarian CHO-K1 cells were purchased from the Korean Cell Line Bank. Under a humidified standard chamber (MCO-19AIC, Sanyo Electric Corp., Osaka, Japan) at 37 °C with 5% CO_2_ atmosphere, the tumor cells were cultured in RPMI-1640 medium containing 10% FBS and 1% penicillin–streptomycin. Prior to the testing, the cells (1 × 10^6^ cells/mL) were harvested using a 0.25% trypsin (*w*/*v*) and 0.03% EDTA solution (*w*/*v*) and then cultured in a well plate [16,23,24,25].

### 2.7. In Vitro Cellular Uptake Studies

Each NP sample (equivalent to TPT 10 μg/mL) or free TPT (10 μg/mL) was incubated with MDA-MB-231 and CHO-K1 cells at pH 7.4 for 4 h. After the cells were washed three times using fresh PBS (150 mM), they were analyzed using the FACSCalibur^TM^ flow cytometer (FACS Canto II, Becton Dickinson, Franklin Lakes, NJ, USA). In addition, the treated cells were visualized using a confocal laser scanning microscope (LSM710, Carl Zeiss, Oberkochen, Germany) [15,31,32,33].

### 2.8. Hemolysis Test

The endosomolytic ability of NPs was determined by conducting a hemolysis analysis [37]. Red blood cells (RBCs) were isolated using fresh mouse blood from BALB/c mice (7-week old female) and purified through centrifugation at 1500 rpm for 10 min three times. The RBCs in the pellet were washed with fresh PBS, and then dispersed in PBS (150 mM, pH 7.4, 6.5, and 6.0). Each NP sample (100 μg/mL) was then incubated with RBC solution (1 × 10^6^ cells/mL) in a shaking water bath at 37 °C for 1 h. The treated RBCs were then centrifuged at 1500 rpm for 10 min, and the light absorbance (LA) of the resulting solution was measured using a microplate reader (Bio-Tek, Winooski, VT, USA) at a 541 nm wavelength. In addition, the RBC solution, which was completely lysed using 2% (*w*/*v*) Triton X-100, was used as a positive control, and the PBS-treated RBC solution was used as a negative control. The endosomolytic activity (%) of NPs was determined from the degree of RBC hemolysis [36,37].

### 2.9. In Vitro Cell Cytotoxicity

The MDA-MB-231 or CHO-K1 cells were incubated with each NP sample (equivalent to TPT 10 μg/mL) and free TPT (10 μg/mL) suspended in RPMI-1640 medium (pH 7.4) at 37 °C for 24 h. The cell viabilities of the treated tumor cells were measured using a CCK-8 assay. In addition, the MDA-MB-231 and CHO-K1 cells were incubated with each NP sample (1–100 μg/mL, without TPT) suspended in an RPMI-1640 medium (pH 7.4) at 37 °C for 24 h to verify the original toxicity of each NP. Furthermore, we evaluated the cell apoptosis of both the MDA-MB-231 and CHO-K1 cells treated with NPs (equivalent to TPT 10 μg/mL) or free TPT (10 μg/mL) at 37 °C for 4 h. These tumor cells were stained with Annexin V–FITC and propidium iodide (PI) for 15 min at 25 °C and then analyzed using the FACSCalibur^TM^ flow cytometer (FACS Canto II, Becton Dickinson, Franklin Lakes, NJ, USA) [15,23,24,25].

### 2.10. Animal Care

In vivo experiments were conducted using 6–8 weeks old female BALB/c mice (Orient Bio Inc., Seoul, Korea). The nude mice were maintained under the guidelines of approved protocol (code: CUK-IACUC-2020-011, 06-08-2020) from the Institutional Animal Care and Use Committee (IACUC) of the Catholic University of Korea (Republic of Korea) [27,28,29,30,31,32,37].

### 2.11. In Vivo Tumor Inhibition

MDA-MB-231 cells (1 × 10^7^ cells/mL in 150 mM PBS, pH 7.4) were subcutaneously administered into the BALB/c nude mice to generate an in vivo xenograft tumor mice model. Subsequently, we intravenously administered an NP sample (with a fluorescent Ce6 dye, equivalent to Ce6 dye 2.5 mg/kg) or free Ce6 dye (2.5 mg/kg) to BALB/c nude mice. The in vivo fluorescent images of the treated mice were captured using a fluorescence-labeled organism bioimaging instrument (FOBI, NeoScience, Suwon, Korea) for 24 h. At 24 h post injection, the treated mice were sacrificed, and the tumor and major organs were harvested and then evaluated using NEOimage software (FOBI, NeoScience, Suwon, Korea) [28,37]. We also intravenously injected NPs (equivalent to TPT 2.5 mg/kg), free TPT (2.5 mg/kg), or saline (control) into the MDA-MB-231 tumor-bearing nude mice. The formula of tumor volume = length × (width)^2^/2 was used to calculate the change in tumor volume over the monitored 7 days [27,28,29,30,31,32,37]. The relative tumor volume change Vt/V_0_ (Vt: tumor volume at a given time, V_0_: initial tumor volume on day 0) was plotted [27,28,29,30,31,32,37].

### 2.12. Statistical Evaluation

All data were analyzed using Student’s *t*-test or analysis of variance (ANOVA) at a significance level of ** *p* < 0.01 [27,28,29,30,31,32,36,37].

## 3. Results and Discussion

### 3.1. Preparation of γCDP-(DMA/PEG-Tf) NPs

To fabricate pH-responsive γCDP-(DMA/PEG-Tf) NPs, we first synthesized γCDP-(DMA/PEG-Tf) (Figure 1b). The carboxylated γCDP (γCDP-COOH) was reacted with ADH and OH-PEG-Mal using DCC, NHS, and DMAP in DMSO solvent and then chemically linked with DMA and Tf, producing multifunctional γCDP-(DMA/PEG-Tf) (Figure 1b). We evaluated the conjugation molar ratios of DMA, PEG, and PA to γCD using proton nuclear magnetic resonance (^1^H-NMR). The ^1^H-NMR results demonstrated that the conjugation molar ratios of DMA, PEG, and PA in γCDP-(DMA/PEG) were 1.0, 0.7, and 1.1 (per one repeating unit of γCD), respectively, which were estimated after analyzing the integration ratio of the peaks from δ 4.61 ppm (–CH– from γCD), δ 1.9 ppm (–CH_3_ from DMA), δ 3.75 ppm (–CH_2_– from PEG), and δ 4.15 ppm (–CH_2_– from PA) (Figure S1, Supplementary Materials). The conjugation molar ratios of PEG and PA in γCDP-(PEG) were 0.7 and 1.1 (per one repeating unit of γCD), respectively, which were estimated after analyzing the integration ratio of the peaks from δ 4.61 ppm (–CH– from γCD), δ 3.75 ppm (–CH_2_– from PEG), and δ 4.15 ppm (–CH_2_– from PA) (Figure S2, Supplementary Materials). We also evaluated the conjugation molar ratios of Ce6 dye to γCDP-(DMA/PEG) (Figure S3, Supplementary Materials) and γCDP-(PEG) (data not shown) using ^1^H-NMR. The ^1^H-NMR results demonstrated that the conjugation molar ratios of Ce6 dye in γCDP-(DMA/PEG) and γCDP-(PEG) were 0.3 and 0.4 (per one repeating unit of γCD), respectively, which were estimated after analyzing the integration ratio of the peaks from δ 4.61 ppm (–CH– from γCD) and δ 2.7 ppm (–CH_2_– from Ce6). Next, the obtained polymers were dialyzed in aqueous solution to fabricate NPs. Then, Tf was chemically tagged to the terminal Mal group of PEG linked to γCDP. As a result, the Tf concentrations (*wt* %) in γCDP-(DMA/PEG-Tf) NPs (or Ce6 dye-tagged γCDP-(DMA/PEG-Tf) NPs) and γCDP-(PEG-Tf) NPs (or Ce6 dye-tagged γCDP-(PEG-Tf) NPs) were 0.40 and 0.44, respectively, which were calculated from the BCA protein assay using free Tf remaining in the supernatant (obtained after the ultracentrifugation of the NPs at 25,000 rpm for 10 min). In addition, the fabrication yield of each NP was 80–90 *wt* %, which was calculated after lyophilization.

We anticipated that γCDP-(DMA/PEG-Tf) NPs with γCD pores (for host–guest interaction), pH-responsive cleavable DMA [22,23], and Tf (for Tf-mediated tumor endocytosis) [2,6,25,26,27] would provide a novel route for multifunctional tumor treatment [37,38,39,40]. Accordingly, we hypothesized that TPT drugs entrapped in the particles endocytosed to Tf-receptor positive tumor cells could be explosively released as a result of ionic repulsion between TPT and DMA-detached γCDP at endosomal pH, which in turn would enable cytosolic TPT release (Figure 1a).

### 3.2. Characterization of γCDP-(DMA/PEG-Tf) NPs

**Figure 2:**
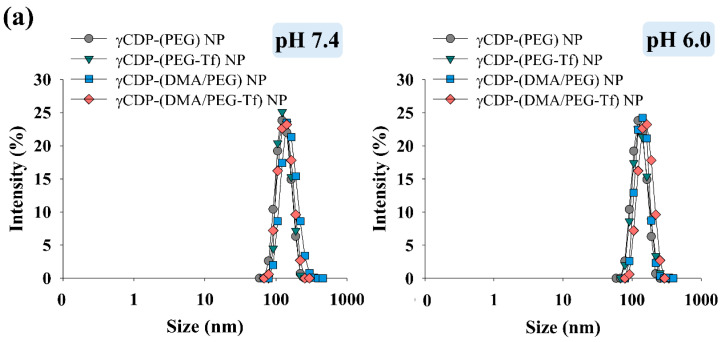

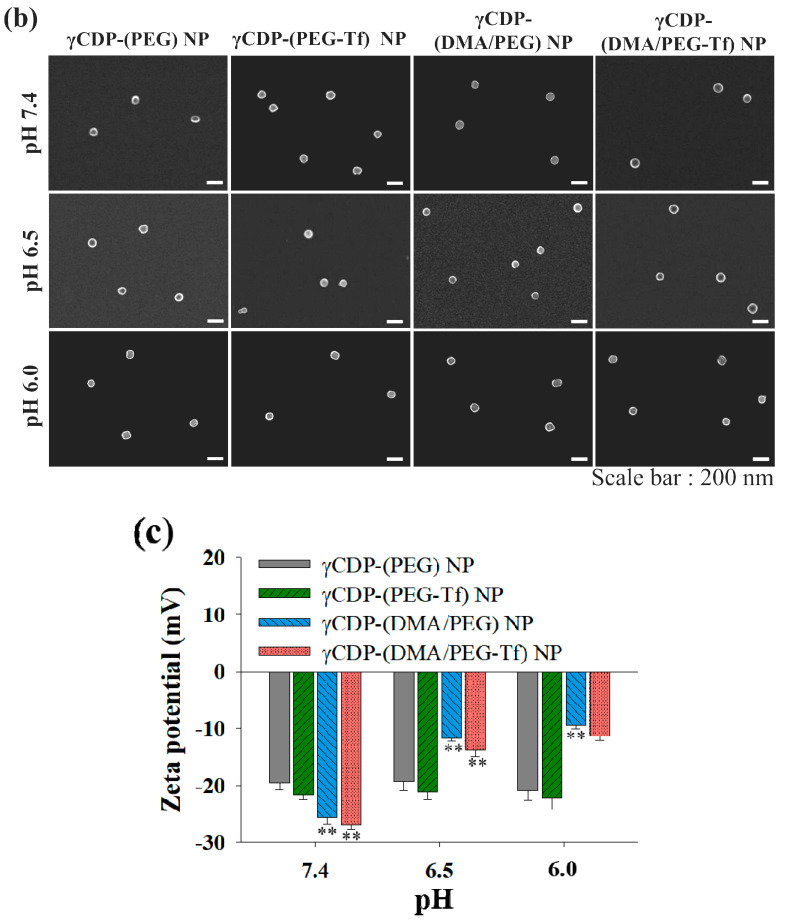
(**a**) Particle size of each sample (0.1 mg/mL) at pH 7.4 and 6.0 (mean ± SD, *n* = 3). (**b**) Field-emission (FE)-SEM images of each sample at pH 7.4, 6.5, and 6.0. (**c**) Zeta potential of each sample (0.1 mg/mL) at pH 7.4, 6.5, and 6.0 (mean ± SD, *n* = 3, ** *p* < 0.01 compared to pH 7.4).

**Figure 3:**
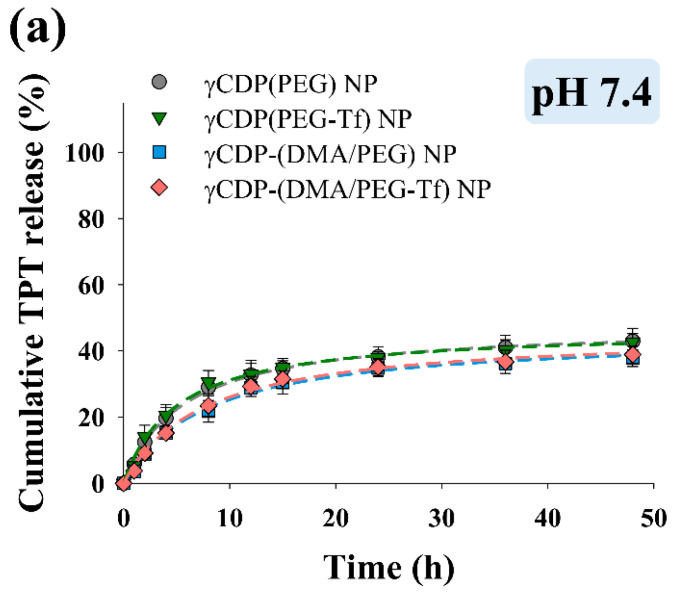

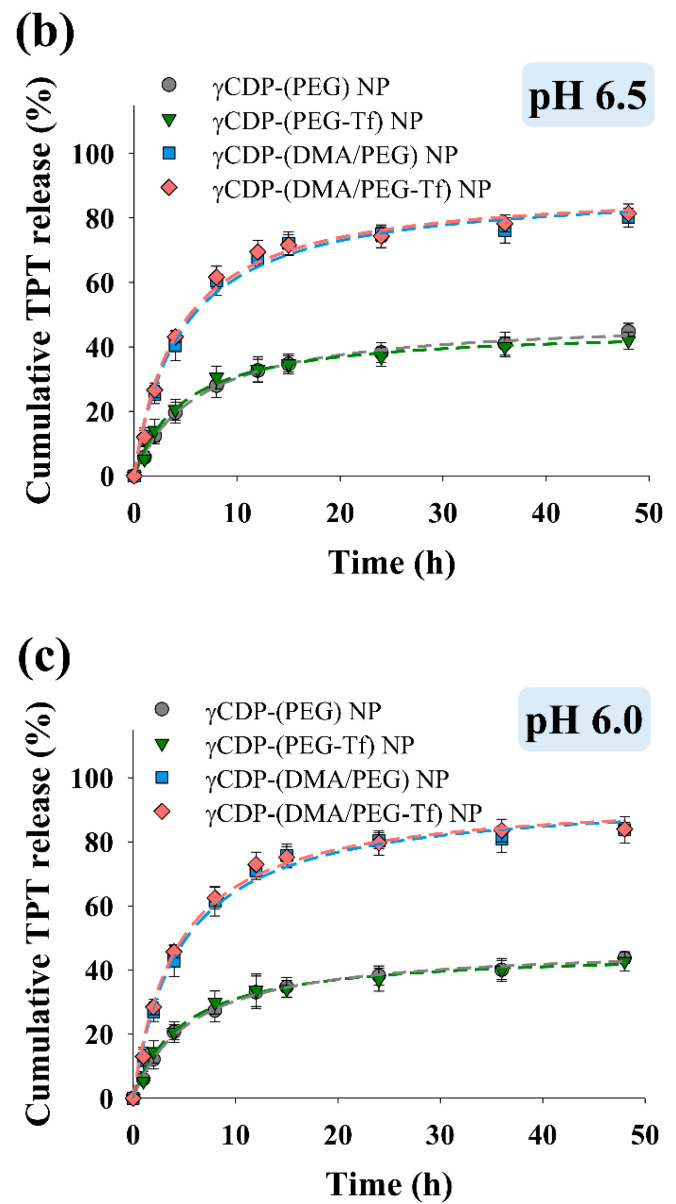
Cumulative topotecan (TPT) release profiles from each sample (equivalent to TPT 1 mg/mL) at (**a**) pH 7.4, (**b**) pH 6.5, and (**c**) pH 6.0 for 48 h (mean ± SD, *n* = 3).

Figure 2a,b show that the average particle sizes of the NP samples ranged from 120 to 134 nm at pH 7.4 and 6.0, indicating that there was no change in particle size according to pH. However, the zeta potential of the γCDP-(DMA/PEG-Tf) NPs increased from −27.6 mV at pH 7.4 to −11.6 mV at pH 6.0 (Figure 2c), most likely due to the cleavage of DMA (backing into cationic primary amine, by hydrolysis) [16,22,23] at pH 6.0, as described in our previous reports. Similarly, the zeta potential of the γCDP-(DMA/PEG) NPs also increased from −24.6 mV at pH 7.4 to −8.4 mV at pH 6.0. However, γCDP-(PEG) NPs and γCDP-(PEG-Tf) NPs without DMA moieties indicated no significant difference in zeta potential when the pH of the solution was reduced to pH 6.0. Consequently, the cleavage of DMA in γCDP-(DMA/PEG-Tf) NPs and γCDP-(DMA/PEG) NPs at pH 6.0–6.5 caused extensive ionic repulsion between γCDP-ADH (without DMA, cationic primary amine) [22,23] and TPT, resulting in the activation of TPT release at pH 6.0–6.5 (Figure 3). In particular, the drug release behaviors of all NPs at pH 7.4 were due to the passive release of TPT from NPs; however, at pH 6.0, the drug release amount of γCDP-(DMA/PEG-Tf) NPs or γCDP-(DMA/PEG) NPs was approximately twofold higher than that of γCDP-(PEG-Tf) NPs or γCDP-(PEG) NPs. Interestingly, within 12 h, the drug release behavior of NPs reached plateaus, and their drug release behaviors up to 12 h showed almost zero-order kinetics. In addition, the *r*^2^ (the correlation coefficient) values of γCDP-(DMA/PEG-Tf) NPs, γCDP-(DMA/PEG) NPs, γCDP-(PEG-Tf) NPs, and γCDP-(PEG) NPs at pH 6.0 were 0.915, 0.921, 0.898, and 0.928, respectively, and the *k* (the zero-order release rate constant) values of γCDP-(DMA/PEG-Tf) NPs, γCDP-(DMA/PEG) NPs, γCDP-(PEG-Tf) NPs, and γCDP-(PEG) NPs were 5.121, 5.109, 2.382, and 2.341, respectively. These results indicate that pH-responsive DMA in γCDP-(DMA/PEG-Tf) NPs mediated the pH-triggered TPT release, probably due to changes in the ionic properties of γCDP pores [15,16,17,22,23].

### 3.3. In Vitro/In Vivo Tumoral Uptake and Tumor Inhibition

**Figure 4:**
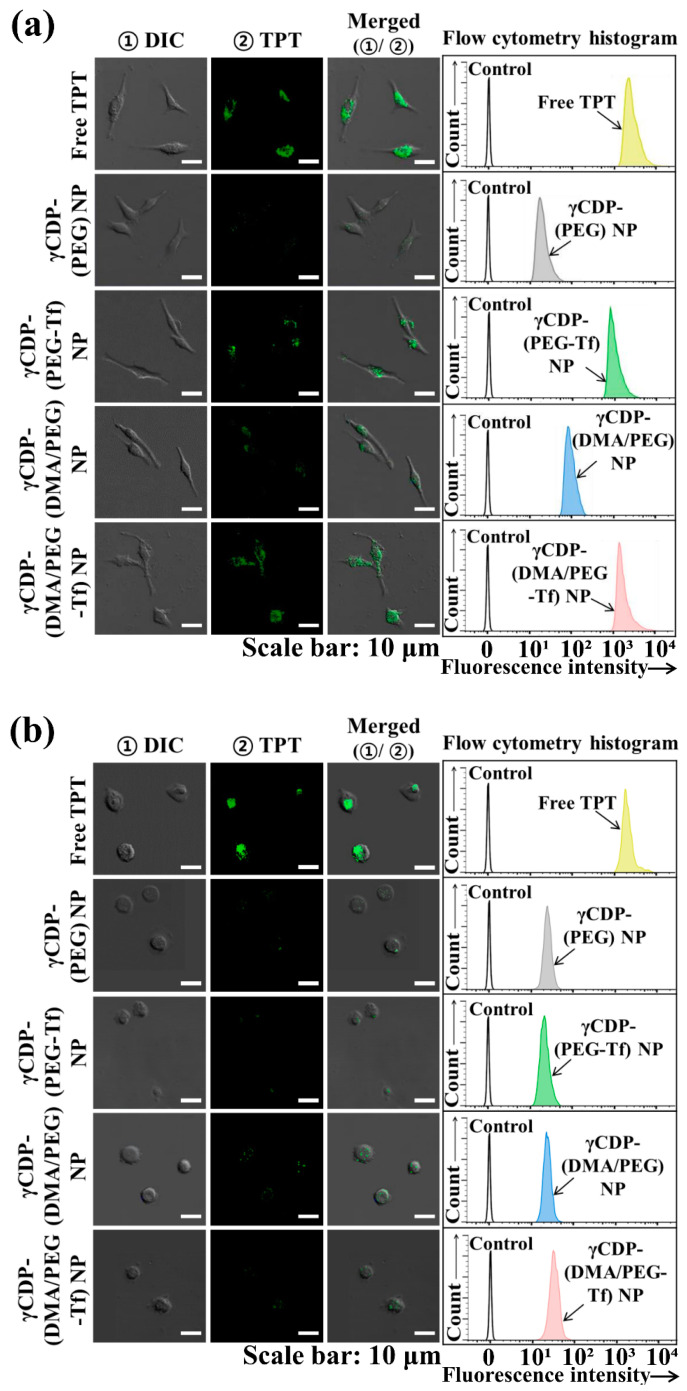
Confocal images and flow cytometry analysis of (**a**) MDA-MB-231 and (**b**) CHO-K1 (as a control) treated with free TPT (10 μg/mL) or each sample (equivalent to TPT 10 μg/mL) for 4 h incubation at 37 °C.

We evaluated the tumor-specific cellular internalization of γCDP-(DMA/PEG-Tf) NPs using confocal microscopy and flow cytometry. Here, MDA-MB-231 cells (Tf receptor-positive) and CHO-K1 cells (as a control, Tf receptor-negative) were treated with fluorescent TPT drug-loaded NP samples (Figure 4). First, the quantitative cellular uptake of TPT-loaded NP samples was measured using a FACSCalibur flow cytometer; the average TPT fluorescence intensities of γCDP-(DMA/PEG-Tf) NPs, γCDP-(DMA/PEG) NPs, γCDP-(PEG-Tf) NPs, γCDP-(PEG) NPs, and free TPT in MDA-MB-231 cells were ~1.9 × 10^3^, ~1.3 × 10^2^, ~1.8 × 10^3^, ~57, and ~2.3 × 10^3^, respectively. Remarkably, γCDP-(DMA/PEG-Tf) NPs, γCDP-(PEG-Tf) NPs, and free TPT presented similar TPT fluorescence intensities to that of MDA-MB-231 cells (Tf receptor-positive) post 4 h incubation (Figure 4a) but displayed different TPT fluorescence intensities compared to that of CHO-K1 cells (Tf receptor-negative) (Figure 4b). Although free TPT with tumor-nonspecific binding properties was highly internalized even to normal cells (CHO-K1 cells), γCDP-(DMA/PEG-Tf) NPs and γCDP-(PEG-Tf) NPs were effective in recognizing MDA-MB-231 tumor cells, presenting their low uptake in normal CHO-K1 cells. Indeed, the TPT fluorescence intensities of γCDP-(DMA/PEG-Tf) NPs and γCDP-(PEG-Tf) NPs in CHO-K1 cells were noticeably shifted to low values (~67 and ~78, respectively). These results indicate that the NPs with Tf ligands were efficiently endocytosed to MDA-MB-231 tumor cells [16,32,33,34,35,36,37], which was also readily apparent in the confocal image results.

**Figure 5:**
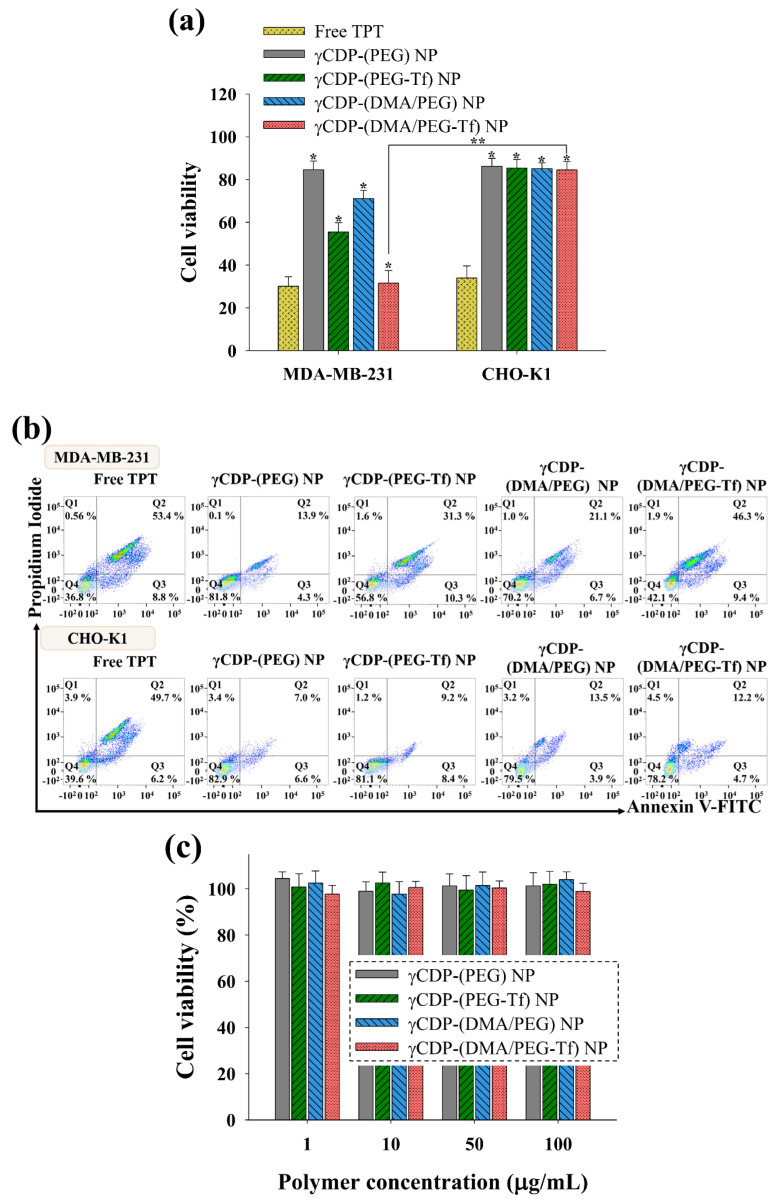
(**a**) In vitro cell viabilities determined by Cell Counting Kit-8 (CCK-8) assay of MDA-MB-231 tumor cells and CHO-K1 cells treated with free TPT (10 μg/mL) or each sample (equivalent to TPT 10 μg/mL) at pH 7.4 for 24 h (*n* = 7); * *p* < 0.01 compared to free TPT and ** *p* < 0.01 compared to γCDP-(DMA/PEG-Tf) NP. (**b**) Flow cytometry analysis of MDA-MB-231 tumor cells and CHO-K1 cells treated with free TPT (10 µg/mL) or each sample (equivalent to TPT 10 µg/mL) at pH 7.4 using Annexin V–fluorescein isothiocyanate (FITC) and propidium iodide (PI). Each quadrant is indicated as follows: Q1, necrotic cells; Q2, late apoptotic cells; Q3, early apoptotic cells; Q4, live cells. (**c**) Cell viability of MDA-MB-231 tumor cells treated with each sample (1–100 μg/mL, without TPT) at pH 7.4 for 24 h (*n* = 7).

Figure 5 shows that TPT-loaded γCDP-(DMA/PEG-Tf) NPs exhibited relatively increased MDA-MB-231 tumor cell death probably as a result of the Tf ligand-mediated endocytosis and endosomal pH-triggered TPT release. In particular, TPT-loaded γCDP-(DMA/PEG-Tf) NPs showed a high cell cytotoxicity, similar to that of free TPT. However, γCDP-(DMA/PEG) NPs without Tf ligands, γCDP-(PEG-Tf) NPs without pH-responsive DMA, and γCDP-(PEG) NPs without both Tf ligands and pH-responsive DMA were less effective in killing MDA-MB-231 tumor cells. Indeed, Figure 5b shows that the apoptotic cell population (Q2 and Q3) [16,30] of MDA-MB-231 cells treated with γCDP-(DMA/PEG-Tf) NPs was 55.7%, but those of MDA-MB-231 cells treated with γCDP-(DMA/PEG) NPs, γCDP-(PEG-Tf) NPs, and γCDP-(PEG) NPs were 27.8%, 41.6%, and 18.2%, respectively. These results were comparable to those of the TPT-loaded NP samples, in which relatively low cell cytotoxicity to normal CHO-K1 cells (Figure 5a), most likely due to their reduced cellular uptake (Figure 4b), was observed. We also evaluated the original toxicity of the NP samples without TPT and found that they had negligible cell cytotoxicity (Figure 5c).

**Figure 6:**
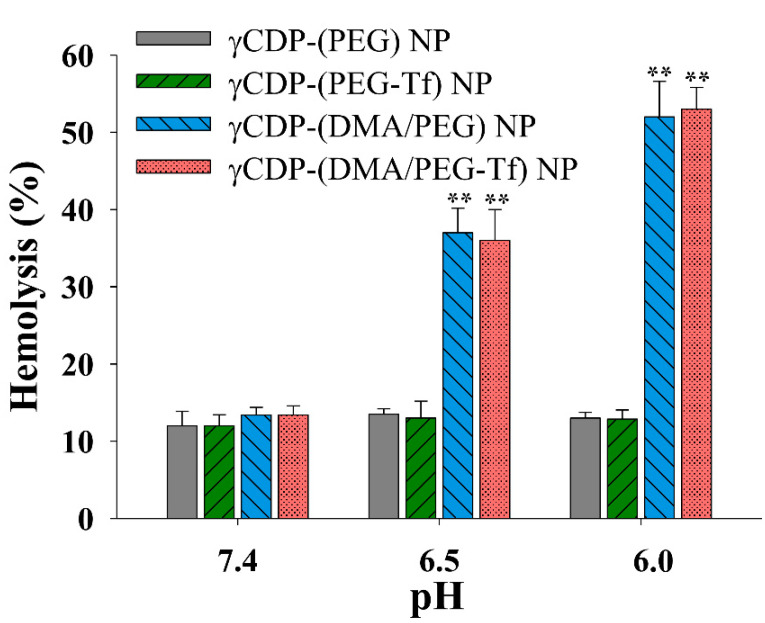
Hemolysis effects of each sample (100 μg/mL) at pH 7.4, 6.5, and 6.0 (n = 3); ** *p* < 0.01 compared to γCDP-(PEG) NP.

Next, we performed a hemolysis experiment using the endosome-like model (i.e., RBCs) to evaluate the endosomolytic activity of γCDP-(DMA/PEG-Tf) NPs (Figure 6). First, at pH 7.4, all NP samples exhibited negligible hemolytic activity [27,37], but γCDP-(DMA/PEG-Tf) NPs and γCDP-(DMA/PEG) NPs presented significantly increased hemolytic activity at pH 6.5 and 6.0 (i.e., endosomal pH), most likely because of the possible proton sponge effect derived from pH-responsive DMA moieties. The cleavage of DMA (resulting in the production of γCDP-ADH backing into cationic primary amine) [22,23] at pH 6.5 could promote the absorption of protons inside endosomes and facilitate cytosolic TPT release from NPs. Indeed, the confocal image of MDA-MB-231 tumor cells treated with γCDP-(DMA/PEG-Tf) NPs showed that TPT was widely distributed, even to the cytoplasm inside the cells (Figure 4a). These results suggest that γCDP-(DMA/PEG-Tf) NPs with Tf ligands and DMA moieties facilitated cytosolic TPT release and enhanced cell cytotoxicity to MDA-MB-231 tumor cells (Figure 5a,b).

**Figure 7:**
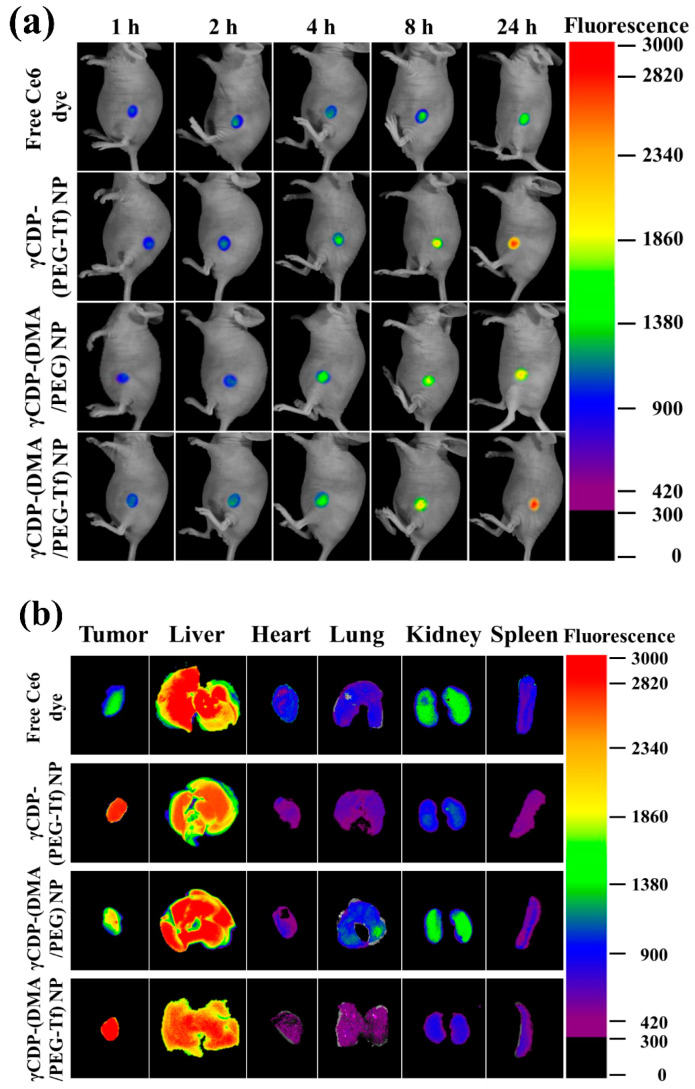

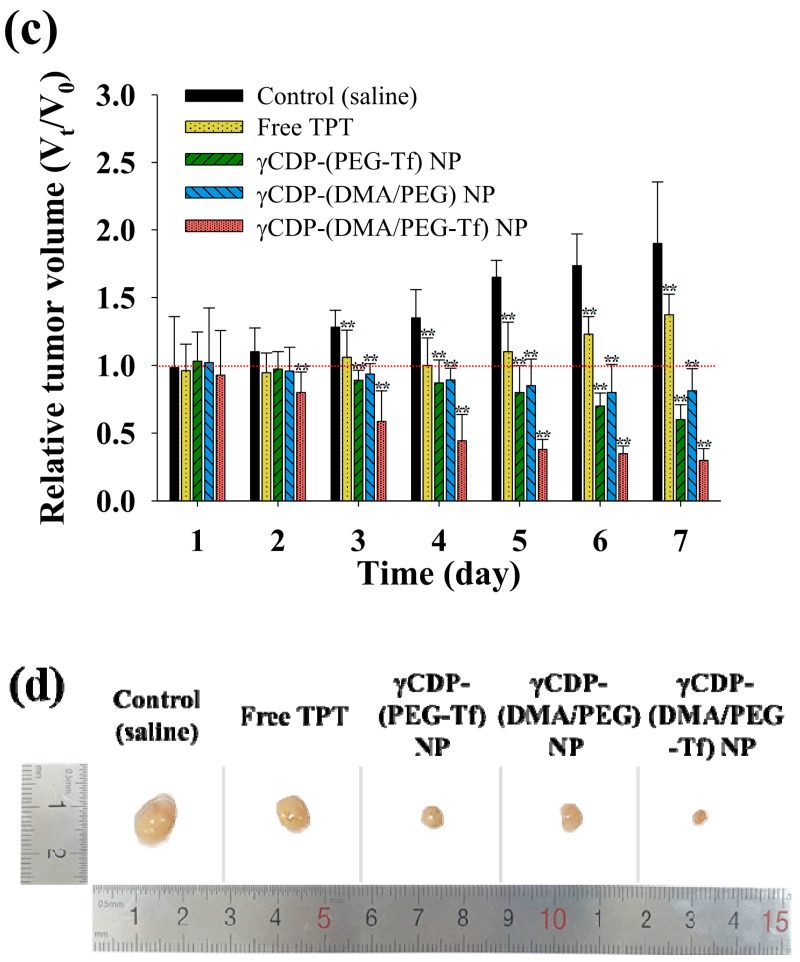
(**a**) Noninvasive in vivo images of free Ce6 dye (2.5 mg/kg) or each Ce6-labeled sample (equivalent to Ce6 dye 2.5 mg/kg, without TPT) intravenously injected into MDA-MB-231 tumor-bearing BALB/c nude mice. Fluorescence images were obtained for 24 h after the injection. (**b**) Ex vivo fluorescence images of tumors and major organs (liver, heart, lung, kidney, and spleen) harvested from MDA-MB-231 tumor-bearing BALB/c nude mice at 24 h post injection. (**c**) Relative MDA-MB-231 tumor volume change (V_t_/V_0_, where V_t_ is the tumor volume at a given time and V_0_ is the initial tumor volume) of MDA-MB-231 tumor-bearing BALB/c nude mice intravenously injected with control (saline), free TPT (2.5 mg/kg), or each sample (equivalent to TPT 2.5 mg/kg) (*n* = 5); ** *p* < 0.01 compared to the control (saline). Red dotted line means 1.0 value. (**d**) Photographs of the tumors excised from the MDA-MB-231 tumor-bearing BALB/c nude mice intravenously injected with control (saline), free TPT (2.5 mg/kg), or each sample (equivalent to TPT 2.5 mg/kg).

In addition, to verify the degree to which the multifunctionality of γCDP-(DMA/PEG-Tf) NPs affected in vivo tumor ablation, we investigated the in vivo pharmaceutical potential of γCDP-(DMA/PEG-Tf) NPs using MDA-MB-231 tumor-bearing BALB/c nude mice [27,28,29,30,31,32,37]. Here, the NP samples were intravenously administered to MDA-MB-231 tumor-bearing BALB/c nude mice, and their fluorescence images and in vivo antitumor efficacy were obtained. Figure 7a,b show that γCDP-(DMA/PEG-Tf) NPs and γCDP-(PEG-Tf) NPs were highly accumulated in the local tumor site, thus supporting their efficient Tf ligand-mediating tumor targeting ability [16,23,24,25,27]. Although the accumulation of γCDP-(DMA/PEG-Tf) NPs and γCDP-(PEG-Tf) NPs in the liver was significant, most likely due to the extensive NP uptake of reticuloendothelial system in the liver [28,37], it was significant that γCDP-(DMA/PEG-Tf) NPs enabled immediate tumor inhibition with the help of the Tf ligands and DMA moieties. The relative tumor volumes (at 7 days post injection) in the nude mice injected with the γCDP-(DMA/PEG-Tf) NPs were approximately 2.8-, 2.1-, 4.6-, and 6.7-fold smaller than those of the nude mice injected with the γCDP-(DMA/PEG) NPs, γCDP-(PEG-Tf) NPs, free TPT, and saline (control), respectively (Figure 7c,d). These results reveal that γCDP-(DMA/PEG-Tf) NPs can preferentially bind to in vivo tumor cells and improve their antitumor activity.

## 4. Conclusions

In this study, γCDP-(DMA/PEG-Tf) NPs was successfully fabricated for highly efficient MDA-MB-231 tumor treatment. In vitro/in vivo results demonstrated that the multifunctionality of γCDP-(DMA/PEG-Tf) NPs enabled increased tumor cell binding affinity and led to significant tumor cell death in vivo. In particular, the destabilization of γCDP pores (resulting from the detachment of DMA moieties at endosomal pH) influenced cytosolic drug release and enhanced cell cytotoxicity. On the basis of the results of this study, we believe that these properties of γCDP-(DMA/PEG-Tf) NPs, prepared using biocompatible and functional materials, can be effective in selectively killing in vivo tumor cells, and there is a high possibility that they will be developed into tumor-targeting nanomedicine with high potential for application to tumor treatment in the future.

## Supplementary Materials

The following are available online at https://www.mdpi.com/1999-4923/12/11/1109/s1: Figure S1. ^1^H-NMR peaks of γCDP-(DMA/PEG); Figure S2. ^1^H-NMR peaks of γCDP-(PEG); Figure S3. ^1^H-NMR peaks of Ce6 dye-tagged γCDP-(DMA/PEG).
