# Supplementary material for: Transferrin-Conjugated pH-Responsive γ-Cyclodextrin Nanoparticles for Antitumoral Topotecan Delivery
Source: Pharmaceutics. 2020 Nov 18;12(11):1109. doi: 10.3390/pharmaceutics12111109 (PMC7698888; doi:10.3390/pharmaceutics12111109)
Supplement: Supplementary file 1 [file pharmaceutics-12-01109-s001.pdf]

Supplementary Materials: Transferrin-Conjugated pH-Responsive γ-Cyclodextrin Nanoparticles for Antitumoral Topotecan Delivery

Seonyoung Yoon, Yoonyoung Kim, Yu Seok Youn, Kyung Taek Oh, Dongin Kim and Eun Seong Lee

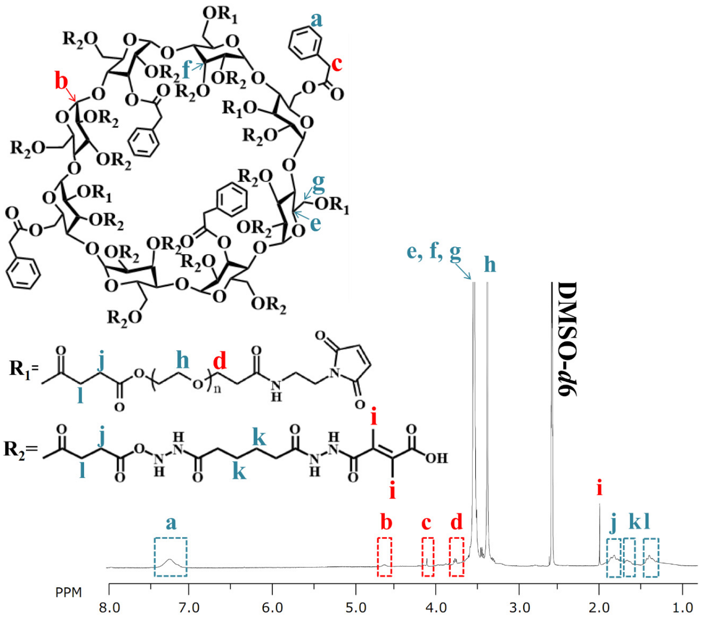

Figure S1. ^{1}H-NMR peaks of γCDP-(DMA/PEG).

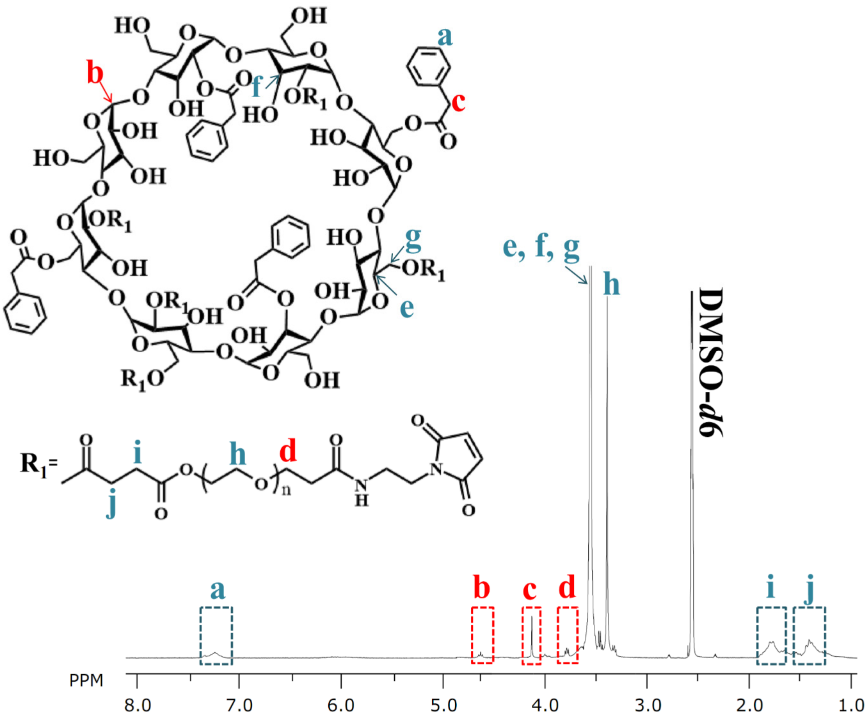

Figure S2. ^{1}H-NMR peaks of γCDP-(PEG).

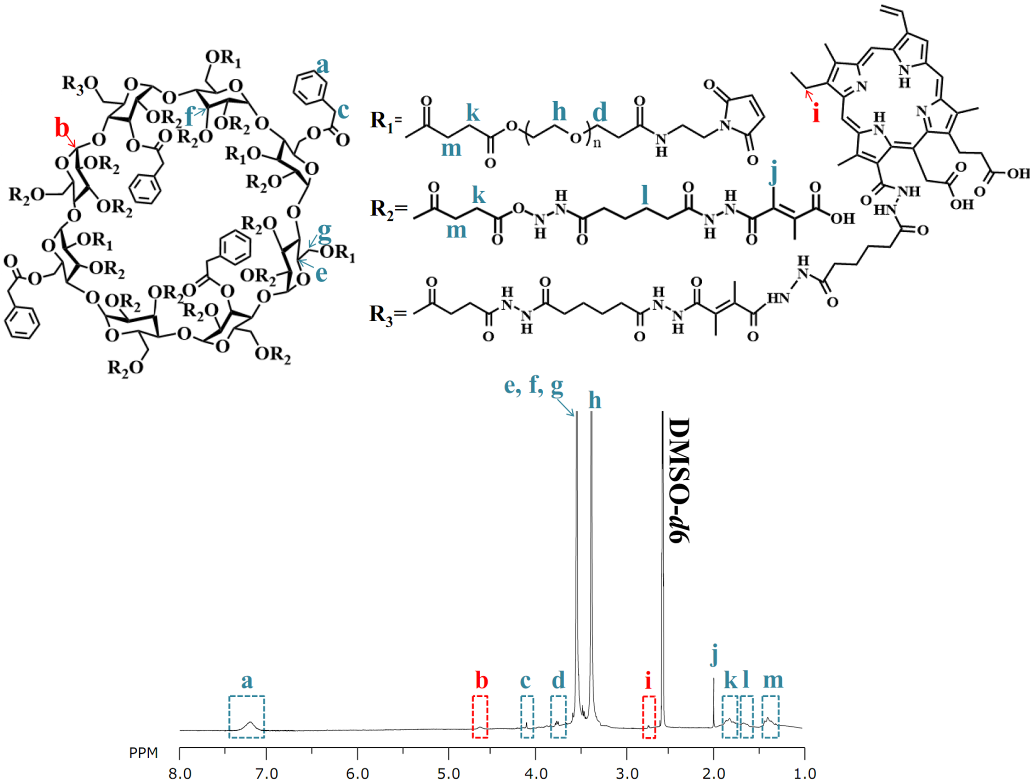

Figure S3. ^{1}H-NMR peaks of Ce6 dye-tagged γCDP-(DMA/PEG).
